# Sensory–Cognitive Profiles in Children with ADHD: Exploring Perceptual–Motor, Auditory, and Oculomotor Function

**DOI:** 10.3390/bioengineering12060621

**Published:** 2025-06-07

**Authors:** Danjela Ibrahimi, Marcos Aviles, Rafael Rojas-Galván, Juvenal Rodríguez Reséndiz

**Affiliations:** 1 Facultad de Medicina, Universidad Autónoma de Querétaro, Santiago de Querétaro 76010, Mexico; danjela.ibrahimi@uaq.mx; 2 Brain Vision & Learning Center, Misión de Capistrano 117, Juriquilla, Santiago de Querétaro 76226, Mexico; 3 Facultad de Ingeniería, Universidad Autónoma de Querétaro, Santiago de Querétaro 76010, Mexico; rafael.rojas@uaq.mx

**Keywords:** ocular treatment, visual health, ADHD, visual therapy, neurostimulation

## Abstract

Objective: This observational cross-sectional study aimed to comprehensively evaluate sensory–cognitive performance in children diagnosed with Attention-Deficit/Hyperactivity Disorder (ADHD), with a focus on auditory processing, visual–perceptual abilities, visual–motor integration, and oculomotor function. The study further examined how hyperactivity, age, and gender may influence these domains. Methods: A total of 70 non-medicated children with clinically diagnosed ADHD (mean age = 9.1±2.4 years; 67.1% male), all with normal visual acuity, were assessed using four standardized instruments: the Test of Auditory Processing Skills, Third Edition (TAPS-3), the Test of Visual Perceptual Skills, Fourth Edition (TVPS-4), the Beery–Buktenica Developmental Test of Visual–Motor Integration, Sixth Edition (VMI-6), and the Developmental Eye Movement (DEM) Test. Statistical analyses included one sample and independent samples *t*-tests, one-way ANOVA, and Pearson correlation coefficients. Results: Participants demonstrated significantly above-average performance in auditory processing (TAPS-3: μ=108.4, std=7.8), average visual–perceptual abilities (TVPS-4: μ=100.9, std=7.2), slightly below-average visual–motor integration (VMI-6: μ=97.1, std=9.0), and marked deficits in oculomotor efficiency (DEM ratio: μ=87.3, std=18.1). Statistically significant differences were observed across these domains (t-values ranging from 2.9 to 7.2, p<0.01). Children with hyperactive-impulsive presentations exhibited lower horizontal DEM scores (μ=73.4, std=12.3) compared to inattentive counterparts (μ=82.9, std=16.2; p=0.009). Age and sex influenced specific subtest scores, with boys and children aged 8–9 years achieving higher outcomes in word memory (p=0.042) and visual discrimination (p=0.034), respectively. Moderate correlations were identified between auditory and visual–perceptual skills (r=0.32, p=0.007), and between visual–perceptual and oculomotor performance (r=0.25, p=0.035). Conclusions: The findings from this sample reveal a distinct sensory–cognitive profile in children with ADHD, characterized by relatively preserved auditory processing and pronounced oculomotor deficits. These results underscore the value of a multimodal assessment protocol that includes oculomotor and visual efficiency evaluations. The conclusions pertain specifically to the cohort studied and should not be generalized to all populations with ADHD without further validation.

## 1. Introduction

This paper evaluates, analyzes, and compares the auditory processing skills, perceptual–motor abilities, and oculomotor patterns of non-medicated children diagnosed with Attention-Deficit/Hyperactivity Disorder (ADHD). The aim is to provide a comprehensive assessment of these sensory modalities to identify which domain is most affected. This knowledge can contribute to the prioritization of evaluations and treatments, ultimately reducing therapy duration and associated costs.

Approximately 6–7% of individuals under 18 are affected by ADHD [1], and the prevalence of the condition has increased significantly in recent years.ADHD is recognized as a neurodevelopmental disorder [2] and is associated with cortical alterations across several brain regions. Studies have identified bilateral reductions in gray matter volume in the early visual cortex, which may contribute to impairments in early-stage attentional mechanisms [3]. Additionally, deficits in visual sensory integration within the occipital cortex, observed in children with ADHD, suggest challenges in early visual information processing [4]. Abnormal activation in the parietal cortex during visual–spatial tasks has been linked to difficulties in visual–perceptual organization and attentional modulation [5].

Recent neuroimaging studies have further advanced our understanding of ADHD’s neurobiological basis. Functional magnetic resonance imaging (fMRI) has revealed reduced activation in the dorsolateral prefrontal cortex during tasks requiring sustained attention, indicating executive dysfunction [6]. Electroencephalography (EEG) findings show elevated theta activity and increased theta/beta ratios in children with ADHD, suggesting cortical underarousal [7]. Event-related potentials (ERPs) demonstrate delayed P300 components, reflecting impairments in attentional resource allocation [8]. In addition, positron emission tomography (PET) studies have shown reduced dopamine transporter availability in the striatum, pointing to altered dopaminergic signaling [9]. These multimodal findings highlight a broad range of neural dysfunctions in ADHD, from electrophysiological to neurochemical and functional domains.

Beyond visual processing, ADHD is also characterized by deficits in visual–motor integration, referring to the ability to coordinate visual input with fine motor output—such as drawing, writing, or copying shapes. Research indicates that children with ADHD perform worse in visual–motor integration tasks compared to typically developing peers [10].

While visual–motor integration reflects everyday coordination between the visual and motor systems, it is important to distinguish it from visuomotor adaptation—a related but conceptually distinct process. The latter refers to the ability to adjust motor responses based on altered or novel visual input. Kurdziel et al. [11] demonstrated impaired visuomotor adaptation in adults with ADHD, including slower learning rates and increased movement variability, associated with cerebellar dysfunction. Structural and functional abnormalities in the cerebellum, particularly in areas governing motor timing and coordination, have been consistently observed [12]. Research indicates that children with ADHD perform worse in visual–motor integration tasks compared to typically developing peers [10].

Oculomotor control is significantly impacted in ADHD. Frontal and prefrontal cortex hypoactivation, particularly in the dorsolateral prefrontal cortex, is associated with executive dysfunction and difficulties in suppressing reflexive saccades, as observed in antisaccade tasks [13,14]. These tasks require individuals to inhibit a natural eye movement (a prosaccade) toward a suddenly appearing visual stimulus and instead voluntarily look in the opposite direction. Performance on antisaccade tasks is therefore considered a sensitive indicator of inhibitory control and prefrontal cortex function. Sherigar et al. [15] demonstrated through forest plot analysis that children with ADHD made significantly more direction errors than controls during these tasks, suggesting impaired oculomotor inhibition. Other oculomotor dysfunctions, such as convergence insufficiency and altered eye movement patterns during reading, have also been observed [16].

Auditory processing deficits are equally prominent in children with ADHD. Kraus and Banai [17] identified impairments in temporal processing and speech sound encoding, linked to atypical activity in the auditory cortex and related neural pathways, which affect language and attention. Disruptions in auditory sequencing and rhythm have been associated with abnormal functioning in the auditory cortex and diminished top-down regulation from prefrontal areas [18,19]. Additionally, studies suggest that children with ADHD exhibit deficits in auditory attention, as reflected by increased omission errors [20] and lower auditory attention quotients [21]. These challenges likely stem from neurodevelopmental factors that impact attention regulation across multiple sensory modalities.

Neuroimaging studies further reveal widespread cortical changes in ADHD, including altered neuronal connectivity. Disruptions in neural circuits responsible for attention and eye movement control have been observed [22]. Functional MRI studies show hyperconnectivity between subcortical structures, such as the caudate, and cortical regions, particularly the prefrontal cortex [23]. Although ADHD primarily affects executive and motivational pathways, these findings suggest broader sensory–cognitive implications.

As can be understood, children with ADHD often present with visual [24] and motor [25] impairments. As a result, these children are frequently referred by psychiatrists and neuropsychologists for neuro-optometric evaluations that go beyond basic visual acuity and refractive assessments. Vision plays a critical role in learning by facilitating the processing, categorization, recognition, and encoding of visual information [26]. Specific areas of difficulty in visual–perceptual functioning include spatial relationships and visual memory [24].

Although many studies have evaluated sensory modalities in isolation, few have simultaneously examined auditory, visual–perceptual, visual–motor, and oculomotor performance in the same group of children with ADHD. Such comprehensive assessments are essential to identify the most impaired domain and to guide the development of specific diagnostic and therapeutic protocols. This approach can shorten therapy duration, reduce costs, and provide actionable insights for health professionals and educators. Teachers, who closely observe these children, would be better equipped to identify early difficulties and refer to them appropriately.

This paper seeks to address this gap by offering a unified evaluation of the sensory systems most often affected in children with ADHD. The purpose of this observational, cross-sectional study is to provide a comprehensive, integrated assessment of auditory processing, visual–perceptual abilities, visual–motor integration, and oculomotor function in children with ADHD, in order to identify the most affected domain and guide future diagnostic and therapeutic strategies.

The structure of this work is as follows: Section 2 details the methods applied, and Section 3 presents the results and findings obtained. In Section 4, the implications and relevance of these findings are discussed. Finally, Section 5 outlines the conclusions drawn and the areas covered within the scope of this work.

## 2. Methods

This section describes the procedures used to conduct the study, including the data selection criteria, testing protocols, and statistical analyses. The research followed a cross-sectional observational design, in which standardized psychometric tests were administered to a single cohort of children diagnosed with ADHD to assess sensory and cognitive performance at one point in time.

**Figure 1:**
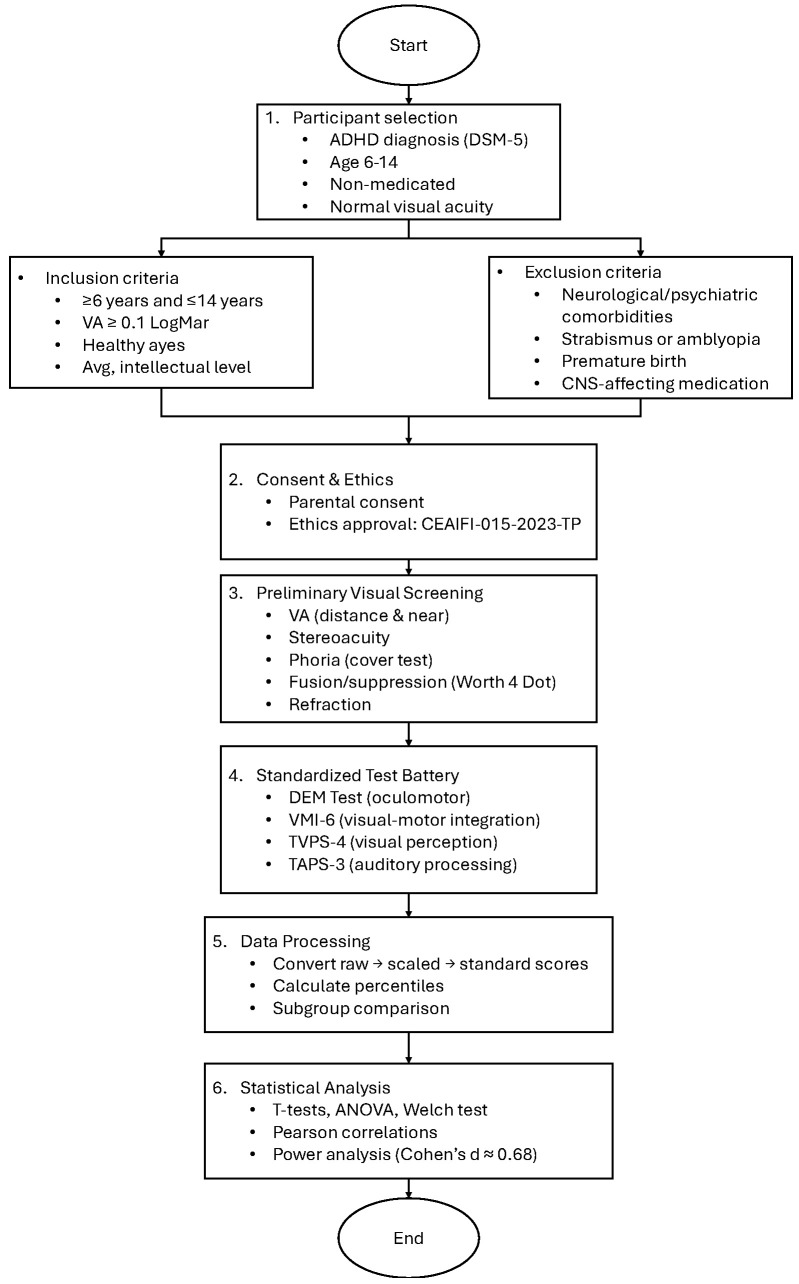
Flowchart of the general methodology followed in this study.

Figure 1 shows the general methodology implemented in this research.

### 2.1. Participants

A total of 70 unmedicated children with ADHD participated in this study. The age range was 6 to 14 years (μ=9.1, std=2.4), with a slight skew toward younger participants. Distribution was approximately even across three subgroups: 6.0–7.11 years (n=27), 8.0–9.11 years (n=17), and 10.0–14.0 years (n=26). The age range was chosen based on clinical guidelines, as ADHD is typically diagnosed after age 6. Although the DEM test is validated up to 13 years and 11 months, all participants were tested before exceeding this age limit, ensuring the psychometric appropriateness of the evaluation tools used. This age range was chosen because ADHD is typically not reliably diagnosed before age 6 [27]. Additionally, most children referred to our department by psychiatrists fall within this range, aligning with clinical recommendations for evaluation. All participants were diagnosed with ADHD by the same psychiatrist according to DSM-5 criteria, which is clinically useful as it allows clinicians to distinguish between children whose primary problems are attention related (inattentive) and those who struggle with hyperactivity-impulsivity [28].

### 2.2. Evaluation Protocol

Evaluations were carried out at the Brain Vision & Learning Center, a diagnostic center for visual problems collaborating with the Autonomous University of Querétaro (UAQ), located at Misión de Capistrano 117, Juriquilla, Querétaro, México. All data were collected by the first author of this work, a professor and researcher at the Faculty of Medicine, UAQ. The study was conducted by the guidelines of the Declaration of Helsinki (Ethics Committee of UAQ, CEAIFI-015-2023-TP approved 1 May 2023), and informed consent was obtained from all parents before any procedure.

All participants underwent a comprehensive evaluation following standardized protocols, consistent with our department’s specialization in assessing, diagnosing, and treating visual dysfunctions. The evaluation consisted of the following steps:

1. Medical history: A detailed medical history was taken for each child.
2. Visual efficiency examination:
  - Motor evaluation: Ocular alignment (phoria state) and eye movement abilities (pursuits and saccades) were evaluated. A cover test at distance and near was used to determine the phoria state of participants and exclude strabismus. Ocular motility was further examined using the NSUCO test to grade pursuits and saccades and to check for any movement limitations (e.g., due to paresis or paralysis).
  - Sensory evaluation: This component assessed visual acuity at distance (6 m) and near (40 cm), stereopsis, binocular fusion, and ocular dominance. Visual acuity was measured with a Bailey–Lovie logMAR chart at both distances to screen for amblyopia. Stereoacuity was measured with the Random Dot 2 test, which progresses from gross (500 arcseconds) to fine (12.5 arcseconds) stereopsis. Flat fusion and suppression were evaluated with the Worth Four Dot test at near, intermediate, and distance, using red–green glasses over the child’s optical correction.
  - Refraction: When needed, children underwent objective and subjective refractions to ensure optimal optical correction for adequate visual acuity.
3. Oculomotor Performance (DEM Test): Oculomotor skills were assessed using the Developmental Eye Movement (DEM) test (version 2.8, 2016), as described by Richman [29]. The DEM test comprises three subtests: two vertical arrays of numbers (Tests A and B) and one horizontal array (Test C). The vertical subtests primarily measure automaticity of number naming, whereas the horizontal subtest evaluates oculomotor function. Each child’s horizontal completion time was adjusted for errors and then divided by the vertical time to calculate a ratio that isolates oculomotor performance from naming automaticity.
  Based on these measures, each child’s DEM performance was categorized into five types:
  - Type 1: Normal performance on all subtests (within average range).
  - Type 2: Abnormally slow horizontal time with relatively normal vertical time, yielding a high horizontal/vertical ratio. This pattern indicates oculomotor dysfunction.
  - Type 3: Slow performance on both horizontal and vertical subtests, but with an average ratio, suggesting an underlying deficit in automaticity (number-naming fluency).
  - Type 4: Abnormal performance on horizontal and vertical subtests as well as an elevated ratio, indicating combined deficits in automaticity and oculomotor control.
  - Type 5: Atypical performance that does not fit Types 1–4. These cases show irregularities in one or more subtests that prevent classification into the standard DEM categories.
4. Visual–Perceptual and Visual–Motor Skills: Visual perceptual skills were evaluated with the Test of Visual–Perceptual Skills, Fourth Edition (TVPS-4), and visual–motor integration was assessed with the Beery–Buktenica Developmental Test of Visual–Motor Integration, Sixth Edition (VMI-6). For both tests, raw scores were converted to derived scores as per their manuals. The TVPS-4 measures seven areas of visual perception: visual discrimination, visual memory, spatial relationships, form constancy, sequential memory, figure–ground, and visual closure. Each TVPS-4 subtest yields a scaled score (mean = 10, Std = 3), and these contribute to an overall standard score (mean = 100, Std = 15) representing general visual–perceptual ability. The Beery VMI-6 provides a measure of fine motor integration; its raw scores were likewise converted to scaled scores and then to standard scores. In this study, a standard score of 85 (20th percentile), which is one standard deviation below the mean, was considered the lower threshold of the normal range for both the TVPS-4 and VMI-6 [30,31]. The scoring procedures for TVPS-4 and VMI-6 were identical, and standardized administration protocols were followed for both tests.
5. Auditory Processing Skills, Third Edition: Spanish/Bilingual Edition (TAPS-3: SBE): Auditory processing abilities were assessed using TAPS-3. This test evaluates skills relevant to everyday listening and learning, through nine subtests that are grouped into three areas:
  - Basic Phonological Skills, e.g., word discrimination, phonological segmentation, phonological blending.
  - Auditory Memory, e.g., number memory forward, number memory reversed, word memory, sentence memory.
  - Auditory Cohesion, e.g., auditory comprehension, auditory reasoning.
  In addition to the standard scores for these areas, the TAPS-3 provides an overall auditory processing score by combining performance across all nine subtests [32].

### 2.3. Test Administration

The study sample comprised children aged 6 to 14 years, categorized into three age groups for analysis: 6–8, 9–11, and 12–14 years. All participants were referred by their treating psychiatrists to undergo a comprehensive neurovisual evaluation as part of an interdisciplinary approach to ADHD assessment. Prior to referral, children had been assessed by other professionals—including otorhinolaryngologists to rule out peripheral auditory impairments and neuropsychologists to support differential diagnosis.

Each participant underwent a standardized 45 min anamnesis conducted by the principal examiner, during which personal and family medical history, developmental milestones, and current behavioral and academic concerns were reviewed. Children presenting with any concomitant neurological, psychiatric, or systemic condition—aside from ADHD—were excluded. Likewise, any child receiving pharmacological treatment at the time of the study was not considered for inclusion. This ensured a homogeneous, non-medicated sample representative of the ADHD population without confounding pathologies.

All children came from mid-to-upper-class families and were enrolled in private educational institutions. These settings offered enriched learning environments with access to specialized academic resources and closer individual monitoring. According to reports from parents and teachers, participants were cognitively capable and intelligent, but they frequently experienced academic difficulties associated with inattention, distractibility, and reduced task persistence. No participants were hospitalized; all assessments were conducted on an outpatient basis in a specialized neuro-optometric clinic.

All evaluations were conducted by the first author, who is a trained clinician and researcher specializing in pediatric neuro-optometric assessment. To ensure consistency, all procedures strictly followed the standardized administration and scoring protocols provided in the respective test manuals. These included step-by-step instructions for administering each subtest, converting raw scores to scaled and standard scores, and interpreting percentile ranks. As only one evaluator conducted all components of the assessment—from initial visual screening to psychometric testing—blinding and inter-rater reliability analyses (e.g., Cohen’s kappa) were not applicable. The use of validated and norm-referenced tools minimized the influence of evaluator bias and ensured scoring reliability across the sample.

### 2.4. Inclusion Criteria

- Age between 6 and 14 years
- Visual acuity ≥0.1 LogMar
- Clinical diagnosis of ADHD with no ongoing medication treatment
- No ocular pathology present (i.e., healthy eyes)
- Average intellectual functioning for age, as reported by the child’s school and psychiatrist

### 2.5. Exclusion Criteria

- Any additional neurological or psychiatric conditions (e.g., epilepsy, depression, or any other neurodevelopmental disorder besides ADHD)
- Primary or secondary strabismus due to trauma, disease, or neurological causes
- Patients with amblyopia
- Use of any medication that could affect central nervous system functioning
- History of premature birth (preterm)

### 2.6. Data Analysis

All test results were scored and analyzed following the standard procedures recommended in each test’s manual. First, each child’s perceptual age (age-equivalent performance level) was determined based on their birth date and the test date. Next, raw scores from each subtest were converted to scaled scores using age-normed tables provided in the test manuals. These scaled scores were then converted to standard scores and percentile ranks to facilitate comparison to normative samples. For tests with multiple subtests that yield a composite score (such as the TVPS-4 and TAPS-3), the scaled scores of the subtests were summed to compute an overall standard score, which was subsequently converted into an overall percentile rank.

### 2.7. Statistical Power Analysis

Statistical analyses included one-way ANOVA, one sample *t*-test, independent *t*-test, Welch test, and Pearson correlations. To make sure the results of this study were meaningful and statistically reliable, a power analysis was conducted after data collection. The calculations were conducted using the TTestIndPower function from the Statsmodels package in Python 3.10.15 which is comparable in accuracy to G*Power and widely used in academic research.

With a total sample of 70 children (divided into two groups for most comparisons), a significance level of 0.05, and a desired power of 80%, this sample size was strong enough to detect a medium-to-large effect size (Cohen’s d ≈ 0.68). In other words, the number of participants was more than adequate to identify relevant differences between groups. A medium effect size typically requires around 64 participants, which means our sample provides confidence in the robustness of the results and the conclusions drawn from them.

## 3. Results

A total of 70 non-medicated and visually normal ADHD patients aged 6 to 14 years participated in this study. The group comprised 67.1% males and 32.8% females, all from similar socio-economic backgrounds (upper-middle class). The mean stereopsis was 29.4 ± 9.8. Among the participants, 81.4% showed exophoria at both distances, far (μ = 0.4 ± 0.9) and near (μ = 8.4 ± 5.2), while 18.5% presented esophoria at both distances (μ = 1.1 ± 1.1 and μ = 3.9 ± 2.1, respectively).

This section is divided into four subsections:

### 3.1. Comparison of Overall Performance Across Standardized Test Scores

**Figure 2:**
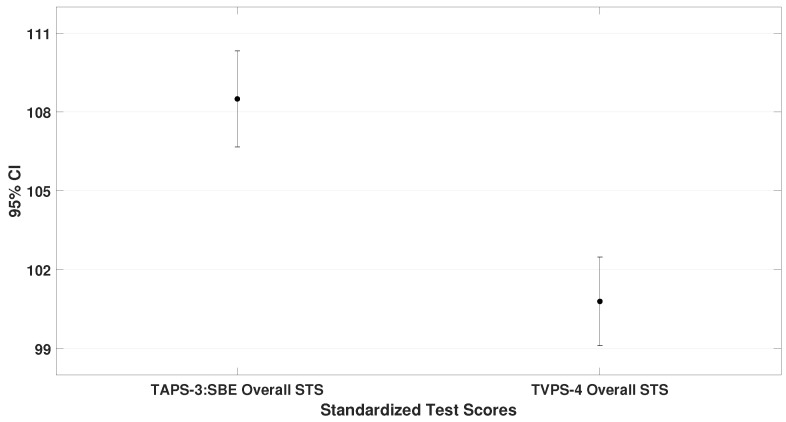
It shows the mean and standard deviations of TAPS-3 (μ = 108.4 ± 7.8) and TVPS-4 (μ = 100.9 ± 7.2). Children with ADHD scored significantly higher on auditory processing skills than on visual–perceptual abilities (t= 7.2, p< 0.001).

**Figure 3:**
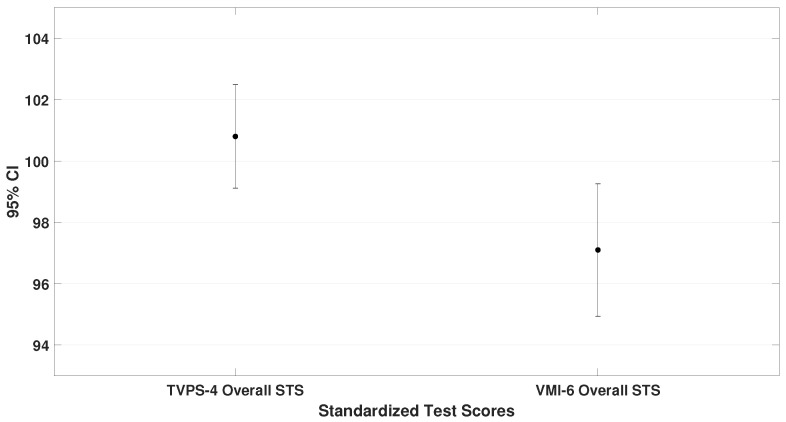
It presents the mean and standard deviation for the TVPS-4 (μ = 100.8 ± 7.2) and VMI-6 (μ = 97.1 ± 9.0). A significant difference was observed, with participants scoring lower on visual–motor integration than on visual–perceptual abilities (t= 2.9, p= 0.005).

**Figure 4:**
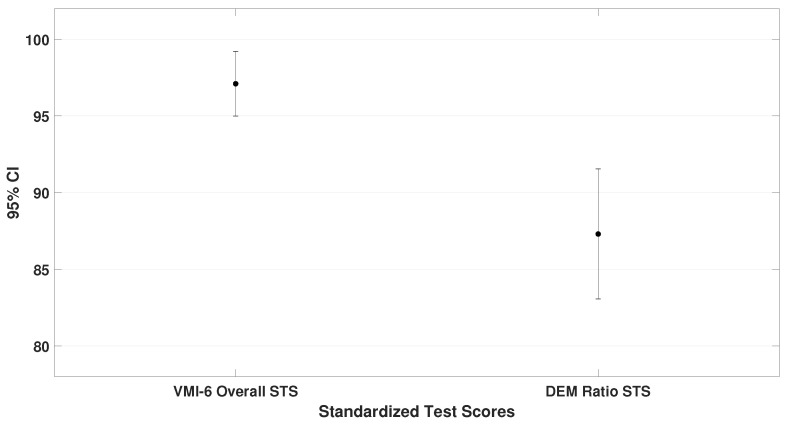
It represents the mean and standard deviation for the VMI-6 (μ = 97.1 ± 9.0) and DEM (μ= 87.3 ± 18.1) test. A significant difference was observed, with participants scoring lower on the DEM than on the VMI-6 test.

This subsection compares the mean standard scores (STS) obtained on the TAPS-3, TVPS-4, VMI-6, and DEM test. These values represent the general performance of the entire sample. Paired-sample *t*-tests were used, and the results are illustrated in Figure 2, Figure 3 and Figure 4.

The oculomotor performance, represented by the DEM ratio (μ = 87.3 ± 18.1), was also analyzed. To demonstrate that children scored lower on the oculomotor test compared to the other assessments, the DEM ratio was specifically compared with the VMI-6 (lowest scores than TAPS-3 and TVPS-4). The analysis showed a significant difference (t= 4.4, p< 0.001), as shown in Figure 4.

Pearson correlation analysis revealed moderate positive correlations at the 0.01 level between TAPS-3 and TVPS-4 (r= 0.32, p= 0.007), and at the 0.05 level between TVPS-4 and DEM ratio (r= 0.25, p= 0.035).

From these data, it can be concluded that children with ADHD performed best on auditory processing tasks and poorest on oculomotor performance (until two standard deviations below mean). Additionally, moderate correlations were found between auditory and visual–perceptual skills, as well as between visual–perceptual and oculomotor performance.

### 3.2. Analysis of Subtest Performance in the DEM, TAPS-3, and TVPS-4 Assessments

This part focused on analyzing the components of the DEM test, TAPS-3, and TVPS-4. For the DEM test, standard scores from the horizontal and vertical subtests were compared using a paired sample *t*-test. No statistically significant differences were found (p= 0.439), although all participants scored until three standard deviations below the mean. Lower scores were observed on the horizontal subtest. Results are shown in Table 1.

Pearson correlation was then performed and a significant relationship at the 0.01 level was found between horizontal and vertical performance, (*r* = 0.67, p< 0.001)

**Figure 5:**
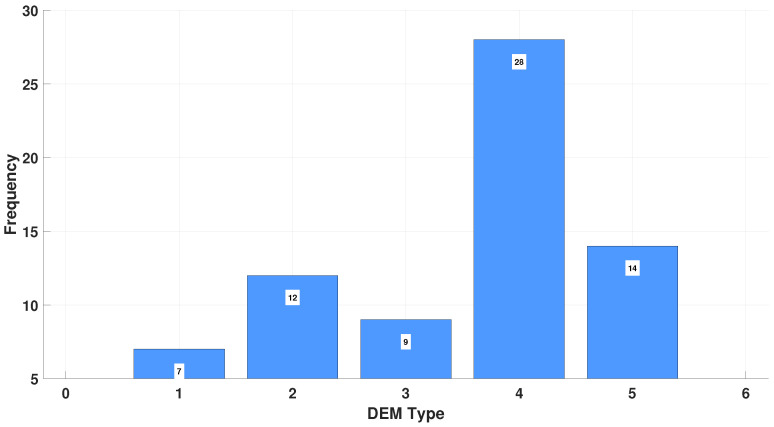
The DEM Type distribution is divided into five categories: Type 1—average performance (10%); Type 2—oculomotor dysfunction (17.1%); Type 3—difficulty with automaticity of number naming (12.9%); Type 4—combined deficits in both automaticity and oculomotor control (40%); and Type 5—uncategorized (20%). As shown in the graph, only 10% of children with ADHD demonstrated average performance, while the remaining 90% exhibited some form of oculomotor dysfunction, with Type 4 being the most prevalent.

As outlined in the methodology, the DEM test categorizes oculomotor performance into five types. Figure 5 shows this distribution: only 10% of ADHD children showed average performance, while 40% presented combined deficiencies in automaticity and oculomotor control (Type 4), the most severe form of oculomotor dysfunctions.

The TAPS-3 test, composed of nine subtests grouped into three main areas (phonological skills, auditory memory, and auditory cohesion), was then analyzed. One sample *t*-tests, paired sample *t*-tests, and Pearson correlations were performed. Recall that standard scores used for the statistical analysis are based on a population distribution having a mean of 100 and standard deviation of 15. Table 2 presents these data.

From Table 2, it can be concluded that children with ADHD scored above the normative mean in all three areas of the TAPS-3 test (p< 0.001), with scores ranging from approximately 0.5 standard deviations below to 1.5 standard deviations above average. Pearson correlation analysis revealed moderate relationships among the three areas, significant at the 0.01 level. The strongest correlation was found between auditory memory and auditory cohesion (r= 0.43, p< 0.001). Overall, children performed best in auditory cohesion, suggesting this is their strongest area of auditory processing.

Analysis of the nine TAPS-3 subtests using one sample *t*-test is presented in Table 3. Just recall that scaled scores are based on a population distribution having a mean of 10 and a standard deviation of 3. Most scores were within the average range. Only two subtests—phonological blending and number memory reversed—were below average but remained within 1 standard deviation, which is considered normal.

Additionally, the paired-samples *t*-test showed no statistically significant difference between these two subtests (p= 0.905), suggesting that both represent the weakest auditory processing skills in children with ADHD. The Pearson correlation between the two lowest-scoring subtests was also not significant (r= 0.20, p= 0.086).

Finally, the seven subtests of the TVPS-4 were analyzed using one sample *t*-test. As shown in Table 4, only sequential memory was below the mean—but again, within 1 standard deviation and not significantly different from the norm.

### 3.3. Group Comparison Between ADHD Inattentive and Hyperactive-Impulsive Subtypes

This subsection compared ADHD inattentive versus hyperactive-impulsive children. A one-way ANOVA showed no statistically significant differences in overall performance for TAPS-3 (p= 0.617), TVPS-4 (p= 0.609), and VMI-6 (p= 0.366). However, the DEM ratio, representing overall oculomotor performance, showed a significant difference between groups (F= 5.5, p= 0.022).

Levene’s test confirmed homogeneity of variances (p= 0.1), and the independent samples *t*-test was applied. Since the DEM ratio is derived from horizontal and vertical subtests, both components were separately analyzed.

For the horizontal subtest, significant group differences were found (F= 6.1, p= 0.016). Levene’s test showed unequal variances (p= 0.041), so the Welch test was used instead. Table 5 summarizes the data.

These results indicate that hyperactivity has a negative impact on the horizontal eye movement performance in children with ADHD.

No significant group differences were found in the three general areas of TAPS-3 or in the nine subtests, nor in the seven subtests of the TVPS-4 as confirmed by one-way ANOVA. Therefore, hyperactivity appears to affect oculomotor function, but not auditory or visual information processing.

### 3.4. Analysis of Sex and Age Differences in Test Performance

This section examined differences based on sex and age, following the same procedures used in the third subsection. A one-way ANOVA was conducted for the initial analysis, and depending on the homogeneity of variances, either the independent samples *t*-test or the Welch test was applied.

#### 3.4.1. Sex Analysis

Levene’s test indicated homogeneity of variances for both the VMI-6 (p= 0.606) and the word memory subtest of TAPS-3 (p= 0.989), so the independent samples *t*-test was used.

**Figure 6:**
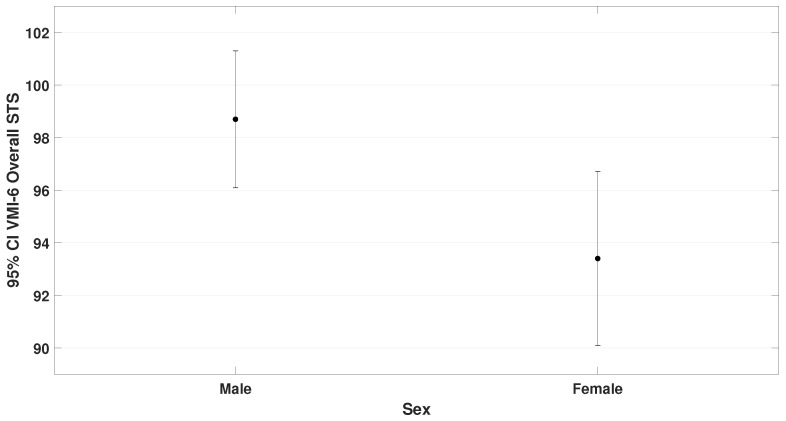
Illustrates the performance differences between sex on the VMI-6 test. Males (μ = 98.8 ± 9.1) performed significantly better than females (μ = 93.6 ± 8.1); t= 2.3, p= 0.023 in the VMI-6 test.

**Figure 7:**
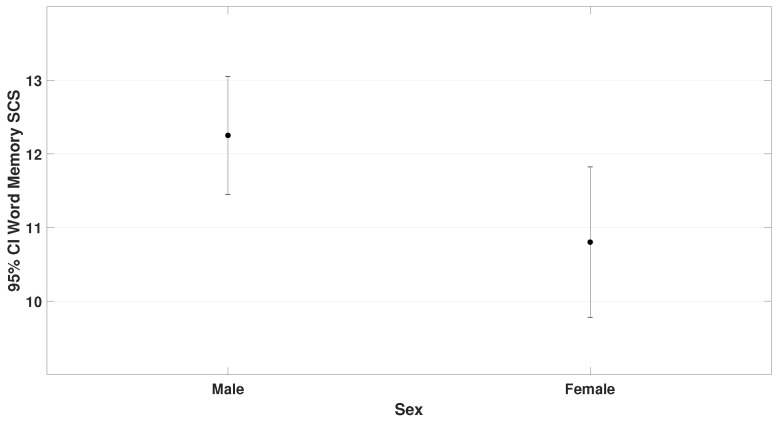
Illustrates sex differences in the word memory subtest of the TAPS-3. Males (μ = 12.2 ± 2.8) scored significantly higher than females (μ = 10.8 ± 2.5); t= 2.1, p= 0.042.

Among the 70 participants, there were 47 males and 23 females. Results showed (F= 5.5, p= 0.023 for VMI-6) and (F= 4.3, *p* = 0.042 for word memory). These findings are presented in Figure 6 and Figure 7.

These findings suggest that male children with ADHD have stronger visual–motor integration and better word recall than females.

#### 3.4.2. Age Group Analysis

Participants were divided into three groups by attention/comprehension level, as well as time spent performing near-distance work:

- Group 1: 6.0–7.11 years (n = 27)
- Group 2: 8.0–9.11 years (n = 17)
- Group 3: 10.0–14.0 years (n = 26)

**Figure 8:**
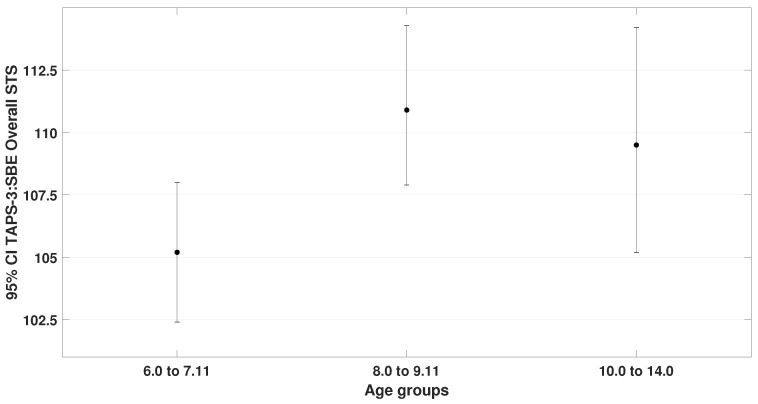
Displays the overall TAPS-3 performance scores across the three age groups. Statistically significant differences were observed only between group 1 (μ = 105.3 ± 7.4) and group 2 (μ = 110.9 ± 7.1), with p= 0.023. Statistically insignificant differences were found when compared to group 3 (μ = 109.5 ± 8.2).

**Figure 9:**
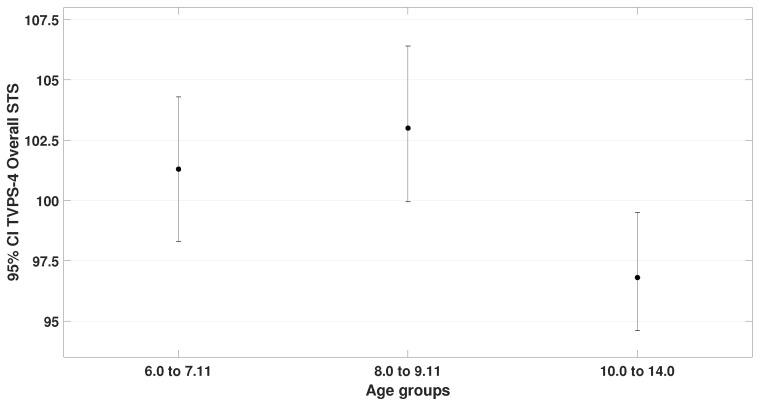
Illustrates the overall performance on TVPS-4 across the three age groups. A statistically significant difference was found between group 2 (μ = 103.0 ± 7.8) and group 3 (μ = 96.8 ± 4.8), p= 0.015. Comparisons with group 1 (μ = 101.3 ± 7.1) did not yield statistically significant results.

One-way ANOVA with Tukey’s HSD (homogeneity of variances by Levene‘s test was confirmed). Significant differences were found for TAPS-3 (F= 3.9, p= 0.025) and TVPS-4 overall performance (F= 4.2, p= 0.019). These results are illustrated in Figure 8 and Figure 9.

No differences were found between age groups in the three general TAPS-3 areas.

However, in the nine subtests, phonological segmentation showed statistical significance (the homogeneity of variances was not met, and the Welch test was used), with group 1 scoring lower than the rest (*t*-statistic = 4.2, p= 0.022). More specifically, these differences were found when group 1 was compared to group 2 and 3. These findings are presented in Table 6.

In TVPS-4 subtests, one-way ANOVA with Tukey’s HSD for multiple comparison found differences in:

- Visual discrimination (F= 5.3, p= 0.008)
- Visual closure (F= 5.1, p= 0.008).

Results are presented in Table 7.

No significant differences were found in DEM horizontal or vertical scores across age groups, suggesting age affects auditory and visual processing, but not oculomotor performance.

## 4. Discussion

This paper analyzed and compared the visual–perceptual and visual–motor integration skills, auditory processing abilities, and oculomotor performance of non-medicated children with ADHD. Although the literature includes a few studies focused on these individual areas, no prior research has conducted such a comprehensive evaluation of these sensory modalities together. This broader approach offers a more integrated understanding of which domains are most affected. By identifying the strengths and weaknesses of children with ADHD, health professionals can better prioritize needs, reducing both therapy duration and associated costs. Additionally, this knowledge can support teachers who often spend most of their time with these children, recognizing their challenges more accurately and refining their observations. Early referral for evaluation can then be encouraged, potentially preventing more serious difficulties later on. As highlighted in recent research, embracing neurodiversity allows for more personalized and effective interventions [33].

In this study, a clear pattern across sensory–cognitive domains in children with ADHD was observed. On average, they performed above the normative mean in auditory processing (TAPS-3), reached near the norm in visual–perceptual skills (TVPS-4), scored moderately below average in visual–motor integration (VMI-6), and demonstrated the lowest performance in oculomotor function (DEM ratio).

Our findings suggest that children with ADHD exhibit a specific profile of sensory strengths and weaknesses, with oculomotor and visual–perceptual domains being more compromised than auditory processing. Neuroimaging findings offer valuable insight into the neural substrates underlying the sensory deficits observed in this study. Functional MRI studies have consistently demonstrated altered activation in the prefrontal cortex, basal ganglia, and cerebellum in individuals with ADHD, particularly during tasks requiring attention regulation and inhibitory control [12,34]. These findings parallel our results, which identified deficits in oculomotor performance and visual–motor integration—skills heavily reliant on frontal and cerebellar circuits. Moreover, electrophysiological studies such as event-related potentials (ERP) and qEEG have reported abnormalities in auditory and visual cortical processing, including delayed P300 latencies and atypical alpha/theta power ratios [35,36]. This aligns with the auditory processing challenges documented in our sample. These converging findings support the notion that functional and structural brain alterations in ADHD manifest as measurable deficits across sensory and motor domains.

This sequence—auditory > visual–perceptual > visual–motor > oculomotor—supports some earlier findings while diverging from others. Notably, the auditory strength seen in our sample aligns with the idea that auditory skills may sometimes be a relative strength in ADHD [17].

Our hypothesis is that consistent linguistic exposure or adaptive listening habits may have contributed to these stronger auditory performances, particularly in tasks involving structured verbal information and memory recall. Meanwhile, the slightly lower performance in visual-sequential memory—though not statistically significant—suggests some difficulties with processing visual sequences. This aligns with the working memory model presented in [26], which outlines the distinction between auditory and visual memory systems. Our findings point toward reduced efficiency in the visual sketchpad, despite a relatively intact phonological loop. Consistent with our results, Redondo et al. [24] observed that ADHD children without comorbid conditions tend to have preserved visual–perceptual skills. Because we screened for visual health and excluded children with learning disorders, it is understandable that our participants showed mostly typical visual–perceptual performance.

The most noticeable challenges were found in oculomotor control. DEM scores, particularly the horizontal subpart and DEM ratio, showed that nearly all participants experienced difficulties with saccadic eye movements. Only 10% of the children performed within the average range, while 90% demonstrated some degree of oculomotor dysfunction. The most prevalent type was a combined deficit in oculomotor control and automaticity (Type 4), seen in 40% of our sample. These findings are consistent with prior studies documenting oculomotor issues in ADHD [13,15,16].

Ababneh et al. [16] reported that 41.9% of children with ADHD showed convergence insufficiency, while controls showed almost none. Mostofsky et al. [13] found that ADHD children made more anti-saccade and anticipatory errors, indicating a lack of inhibition and poor control over eye movements. Sherigar et al. [15] also emphasized deficits in voluntary saccades, which supports our finding that ADHD children face challenges in executing controlled saccadic eye movements required in the DEM.

Interestingly, although the DEM and VMI-6 both involve visuomotor coordination, our participants scored notably lower on the DEM. This suggests that managing saccadic eye movements under time pressure (as in reading) may be more difficult for them than general hand–eye coordination required during the copy of images. Earlier research found that children with ADHD had more intrusive saccades and unstable fixations, which mirrors the behavior we observed during the DEM [14].

Taken together, our results emphasize the importance of context and task type. Simões et al. [20] found that ADHD children showed greater impairment on auditory continuous performance tests than on visual ones. However, their auditory tasks lacked meaningful interaction, unlike the socially engaging and structured auditory tasks in the TAPS-3. This supports the idea proposed by Kraus and Banai that auditory processing can change depending on experience [17], and also reflects the view that ADHD performance is heavily influenced by task context [18].

### 4.1. Subtest Analysis, Subgroup Comparisons, and Demographic Effects

In addition to analyzing the overall performance across the four tests, we examined the performance of individual subtests within each standardized assessment. We also explored the impact of demographic and behavioral factors, including the presence or absence of hyperactivity, and the influence of age and sex.

Results showed that horizontal DEM scores were slightly lower than vertical scores, both well below the norm. This pattern, supported by Groß et al. [19], reflects particular difficulty with saccadic eye movements involved in reading. Forty percent of our ADHD children were classified under the most severe DEM type, pointing to a mixed deficit in saccadic eye movements and naming speed. This aligns with Nazari et al. [4], who suggested that ADHD children may show reduced task engagement and automaticity.

Nevertheless, all three TAPS-3 auditory subtests showed above-average scores, suggesting that auditory processing may be an area of strength or compensation in ADHD. On the other hand, in TVPS-4, scores were mostly average or higher, except for sequential memory. This finding is consistent with studies highlighting ADHD-related challenges with planning and memory of sequences tied to working memory limitations and executive function deficits, both common in ADHD [25]. Early research showed differences in occipital structures among adults with ADHD, which could explain subtle issues in their visual processing [3].

### 4.2. Hyperactivity Subgroup Differences

Children with hyperactive traits performed significantly worse on the DEM test, particularly in horizontal scanning, compared to those without hyperactivity. However, there were no significant differences in TAPS-3, TVPS-4, or VMI-6 scores between the subgroups. These results suggest that hyperactivity exacerbates oculomotor challenges, while core auditory and visual–perceptual abilities remain relatively consistent across ADHD types. This finding aligns with previous research, including Biederman et al. [37], Martinussen et al. [38], and Kaufman et al. [39], which have reported similar levels of performance across ADHD subtypes in auditory processing, visual–perceptual skills, and visual–motor integration tasks, respectively. When it comes to eye movements control, [5] linked hyperactivity to reduced volumes in the ventral striatum, which may affect inhibition and movement control. Our findings also relate to those of Hanisch et al. [14], who observed that children with ADHD show poor oculomotor inhibition and unstable fixation, and of Cubillo et al. [22], who emphasized that motor control difficulties in hyperactive ADHD subtypes may arise from fronto-striatal and cerebellar dysfunctions—both of which align with the impaired DEM performance in our hyperactive group.

### 4.3. Age and Sex Effects

Our gender analysis revealed that boys performed slightly better than girls in VMI-6 and word memory tasks of TAPS-3. This could reflect differences in activity patterns, as boys may have more opportunities to practice visuomotor skills which suggests that greater engagement in play and physical activities may help boys strengthen their visuomotor integration skills [25]. Regarding age, children aged 8–9 outperformed younger peers in TAPS-3 and older ones in TVPS-4. This may reflect developmental peaks in auditory and visual skills. Uddin et al. [40] discussed age-related brain network reorganization during attention tasks. He demonstrated that functional connectivity in attention networks reorganizes dynamically during middle childhood, supporting age related gains in auditory attention and working memory. On the other hand, Kraus and Banai [17] emphasized the malleability of auditory pathways—through language exposure and musical training—which may underlie the peak in auditory processing at this age. Alternatively, the demands of TVPS 4 increase for older children, so performance may dip if task complexity outpaces individual development.

Although all participants in this study came from similar sociocultural backgrounds—namely, upper-middle-class families with access to private education and enriched learning environments—subtle influences related to age and sex may still reflect internalized gender norms and culturally mediated developmental expectations. For example, even in highly stimulated environments, boys may be more encouraged to engage in physical, spatial, or action-oriented play, which could support the development of visual–motor coordination. In contrast, girls might receive greater reinforcement for verbal, organized, or sedentary activities, possibly influencing auditory processing or sequential memory tasks [41].

Parental expectations and classroom dynamics in academically demanding settings may also play a role. Teachers and caregivers in such environments may unknowingly adjust their communication or behavioral expectations based on the child’s sex, thereby shaping their engagement with attention-driven or sensorimotor activities. Additionally, subtle peer dynamics within structured educational contexts could influence self-regulation, attention deployment, and task persistence differently across sexes and developmental stages [42].

These sociocultural micro-factors—operating even within a privileged and homogeneous group—may contribute to the age- and sex-related differences observed in this study and merit consideration in future neurodevelopmental research.

These results highlight the necessity of a multimodal approach in both assessment and intervention. In-depth evaluation of the visual system and its components should be prioritized, especially considering that during development, the visual system initially demands substantial cortical resources. Over time, these functions are expected to become automatized; however, when this automation does not occur, visual tasks continue to consume cognitive energy that should instead support higher-order processes, such as those required for executive functioning.

As a result, learning may be compromised, and academic difficulties may emerge and persist. This view is supported by Gori and Facoetti [43], who emphasized that inefficiencies in visual–spatial attention can hinder reading and learning, and by Stein et al. [44], who noted that when visual processing remains effortful, it can divert cognitive resources away from executive functioning. Birch and Saunders [45] also highlighted that early delays in visual development can have cascading effects on later academic performance. For this reason, detailed consideration of visual performance should be incorporated into ADHD assessments to better support academic development and learning outcomes.

## 5. Conclusions

Our findings reveal a distinct ADHD profile characterized by strong auditory processing, mild challenges in visual sequencing, underdeveloped visual-integration abilities, and pronounced oculomotor deficits—particularly among children with hyperactivity. These results underscore the heterogeneous nature of ADHD and the need for a nuanced understanding of its manifestations across sensory domains.

To optimize intervention strategies, we propose a structured and hierarchical assessment approach:

1. Evaluation of the visual system efficiency and sensorimotor integration
2. Examination of oculomotor control—with particular emphasis on saccadic eye movements
3. Assessment of visual–motor integration
4. Analysis of visual–perceptual functioning
5. Investigation of auditory processing skills

A comprehensive understanding of the interdependence between sensory, cognitive, and behavioral systems enables more accurate identification of each child’s unique profile and supports the development of personalized treatment strategies tailored to their developmental needs. Such insight holds the potential to enhance functional outcomes, academic achievement, and long-term quality of life.

## Figures and Tables

**Table 1:** Depicts the scores of the DEM test. ADHD children performed similarly in both subtests, with results from average to three standard deviations below average. Lower performance was observed during horizontal text reading.

DEM Test	Mean Value	Std	*t*-Value	*p*-Value
Horizontal STS	79.8	15.6	−0.8	0.439
Vertical STS	81.1	17.5		

STS: standard scores; Std: standard deviation.

**Table 2:** Represents the statistical analysis of the three general areas of TAPS-3. ADHD children scored above average in all three TAPS-3 areas, with moderate correlations—particularly between auditory memory and cohesion.

Areas of TAPS-3	One Sample *t*-Test					
	Mean ± Std	*p*-Value				
Phonological skills	108.2 ± 10.1	p<0.001				
Auditory memory	105.3 ± 9.8	p<0.001				
Auditory cohesion	116.1 ± 10.9	p<0.001				
	**Paired Sample *t*-Test**		**Pearson analysis correlation**			
	*t*-value	*p*-value		*r*-value		*p*-value
Phonological skills and auditory memory	2.2	0.034		0.37		0.002
Phonological and auditory cohesion	−5.4	<0.001		0.38		0.006
Auditory memory and cohesion	−8.1	<0.001		0.43		<0.001

**Table 3:** This table presents the mean values, standard deviations, and deviations from the norm for all nine subtests of the TAPS-3. As shown in the data, the only two subtests with scores below the average are phonological blending and number memory reversed. However, these differences are not statistically significant when compared to the normative population.

Subtests of the TAPS-3	Mean	Std	*p*-Value
Word Discrimination	13.9	2.1	0.001
Phonological Segmentation	11.1	3.4	0.007
Phonological Blending	9.7	2.4	0.326
Number Memory Forward	10.5	2.2	0.072
Number Memory Reversed	9.7	2.3	0.243
Word Memory	11.7	2.8	0.001
Sentence Memory	12.3	2.4	0.001
Auditory Comprehension	14.6	2.9	0.001
Auditory Reasoning	11.7	2.1	0.001

Std: standard deviation.

**Table 4:** Presents the mean values, standard deviations, and deviations from the norm for all seven subtests of the TVPS-4. As shown in the data, the only subtest with a score below average is sequential memory. However, this difference is not statistically significant when compared to the normative population.

Subtests of the TVPS-4	Mean	Std	*p*-Value
Visual Discrimination	10.3	2.4	0.378
Visual Memory	10.0	2.3	1.0
Spatial Relationship	10.3	2.1	0.316
Form Constancy	10.4	2.5	0.155
Sequential Memory	9.7	2.5	0.275
Visual Figure-Ground	10.4	2.5	0.243
Visual Closure	10.1	2.2	0.826

Std: standard deviation.

**Table 5:** Shows DEM ratio and horizontal subtest results in ADHD inattentive and hyperactive-impulsive children. Inattentive children performed from 0.5 above to 2 standard deviations below mean, while children with hyperactivity-impulsivity scored approximately from 0.5 to 3 standard deviations below mean, indicating severe oculomotor dysfunction.

Independent Sample *t*-Test	Diagnosis	N	Mean ± Std	*t*-Value	*p*-Value
DEM ratio	ADHD inattentive	47	90.7 ± 18.8	2.3	0.022
	ADHD hyperactive	23	80.3 ± 14.4		
**Welch Test**				***t*-statistic**	***p*-value**
DEM Horizontal	ADHD inattentive	47	82.9 ± 16.2	7.4	0.009
	ADHD hyperactive	23	73.4 ± 12.3		

**Table 6:** Presents the results for the phonological subtests and highlights differences among age groups. The data indicate that the youngest group (group 1) obtained the lowest scores, while the oldest group (group 3) achieved the highest performance levels.

Group	Phonological Segmentation (Mean ± Std)	Compared Groups *p*-Value
Group 1	9.6 ± 4.2	1 vs. 2 p= 0.038
Group 2	11.9 ± 2.2	1 vs. 3 p= 0.024
Group 3	12.4 ± 2.5	

Std: standard deviation.

**Table 7:** Presents the two key variables of statistical interest for the TVPS-4: visual discrimination and visual closure. The findings suggest that the oldest group (group 3) scored lower on both subtests, whereas the second group (group 2) achieved the highest scores.

Group	Visual Discrimination (Mean ± Std)	Compared Groups	Visual Closure (Mean ± Std)	Compared Groups *p*-Value
Group 1	9.8 ± 2.5	1 vs. 2 p= 0.034	10.1 ± 2.1	
Group 2	11.4 ± 2.3	2 vs. 3 p= 0.013	10.8 ± 2.3	2 vs. 3 p= 0.006
Group 3	9.3 ± 1.8		8.8 ± 1.5	

Std: standard deviation.

## Data Availability

The database is available upon request to the authors.

## References

[1] Salari N., Ghasemi H., Abdoli N., Rahmani A., Shiri M.H., Hashemian A.H., Akbari H., Mohammadi M. (2023). The global prevalence of ADHD in children and adolescents: A systematic review and meta-analysis. Ital. J. Pediatr..

[2] Sonuga-Barke E.J.S., Becker S.P., Bölte S., Castellanos F.X., Franke B., Newcorn J.H., Vitiello B. (2023). ADHD and the neurodevelopmental paradox: A neurobiological model of functional connectivity in ADHD. Biol. Psychiatry.

[3] Ahrendts J., Rüsch N., Wilke M., Philipsen A., Eickhoff S.B., Glauche V., Perlov E., Ebert D., Hennig J., Tebartz van Elst L. (2011). Visual cortex abnormalities in adults with ADHD: A structural MRI study. World J. Biol. Psychiatry.

[4] Nazari M.A., Berquin P., Missonnier P., Aarabi A., Debatisse D., De Broca A., Wallois F. (2010). Visual sensory processing deficit in the occipital region in children with attention-deficit/hyperactivity disorder as revealed by event-related potentials during cued continuous performance test. Neurophysiol. Clin..

[5] Carmona S., Vilarroya O., Bielsa A., Tremols V., Soliva J.C., Rovira M., Tomas J., Raheb C., Gispert J.D., Batlle S. (2005). Global and regional gray matter reductions in ADHD: A voxel-based morphometric study. Neurosci. Lett..

[6] Bush G., Valera E.M., Seidman L.J. (2005). Functional neuroimaging of attention-deficit/hyperactivity disorder: A review and suggested future directions. Biol. Psychiatry.

[7] Barry R.J., Clarke A.R., Johnstone S.J. (2003). A review of electrophysiology in attention-deficit/hyperactivity disorder: I. Qualitative and quantitative electroencephalography. Clin. Neurophysiol..

[8] Johnstone S.J., Barry R.J., Clarke A.R. (2013). Ten years on: A follow-up review of ERP research in attention-deficit/hyperactivity disorder. Clin. Neurophysiol..

[9] Volkow N.D., Wang G.J., Kollins S.H., Wigal T.L., Newcorn J.H., Telang F., Fowler J.S., Zhu W., Logan J., Ma Y. (2009). Evaluating dopamine reward pathway in ADHD: Clinical implications. JAMA.

[10] He H., Gao X., Liu F. (2022). Characteristics and associated factors of visual and motor integration in children with developmental dyslexia and attention deficit hyperactivity disorder. Neuropsychiatr. Dis. Treat..

[11] Kurdziel L.B.F., Dempsey K., Zahara M., Valera E., Spencer R.M.C. (2015). Impaired visuomotor adaptation in adults with ADHD. Exp. Brain Res..

[12] Valera E.M., Faraone S.V., Murray K.E., Seidman L.J. (2007). Meta-analysis of structural imaging findings in attention-deficit/hyperactivity disorder. Biol. Psychiatry.

[13] Mostofsky S.H., Lasker A.G., Cutting L.E., Denckla M.B., Zee D.S. (2001). Oculomotor abnormalities in attention deficit hyperactivity disorder: A preliminary study. Neurology.

[14] Hanisch C., Radach R., Holtkamp K., Herpertz-Dahlmann B., Konrad K. (2006). Oculomotor inhibition in children with and without attention-deficit hyperactivity disorder (ADHD). J. Neural Transm..

[15] Sherigar S.S., Gamsa A.H., Srinivasan K. (2023). Oculomotor deficits in ADHD: A meta-analysis. Eye.

[16] Ababneh L.T., Bashtawi M., Ababneh B.F., Mahmoud I.H., Rashdan M., Zahran M. (2020). Ocular findings in children with ADHD: A case–control study. Ann. Med. Surg..

[17] Kraus N., Banai K. (2007). Auditory-processing malleability: Focus on language and music. Curr. Dir. Psychol. Sci..

[18] Slater J.L., Tate M.C. (2018). Timing deficits in ADHD: Insights from the neuroscience of musical rhythm. Front. Comput. Neurosci..

[19] Groß C., Serrallach B.L., Möhler E., Pousson J.E., Christiner M., Schneider P., Bernhofs V. (2022). Neuromorphological and neurofunctional correlates of ADHD and ADD in the auditory cortex. Front. Neurosci..

[20] Simões E.N., Carvalho A.L.N., Schmidt S.L. (2021). Role of visual and auditory stimuli in continuous performance tests. J. Atten. Disord..

[21] Wei Z., Yang S., Zhuang D., He X. (2024). Auditory attention deficits in children with ADHD: Evidence from auditory attention quotient and electrophysiological markers. Neuropsychology.

[22] Cubillo A., Halari R., Smith A., Taylor E., Rubia K. (2012). A review of fronto-striatal and fronto-cortical brain abnormalities in children and adults with ADHD and new evidence for dysfunction in adults with ADHD during motivation and attention. Cortex.

[23] Uddin L.Q., Supekar K., Ryali S., Menon V. (2011). Dynamic Reconfiguration of Structural and Functional Connectivity Across Core Neurocognitive Brain Networks with Development. J. Neurosci..

[24] Redondo B., Molina R., Cano-Rodríguez A., Vera J., García J.A., Muñoz-Hoyos A., Jiménez R. (2019). Visual perceptual skills in attention-deficit/hyperactivity disorder children: The mediating role of comorbidities. Optom. Vis. Sci..

[25] Mokobane M., Pillay B.J., Meyer A. (2019). Fine motor deficits and attention deficit hyperactivity disorder in primary school children. S. Afr. J. Psychiatry.

[26] Baddeley A. (2000). The episodic buffer: A new component of working memory?. Trends Cogn. Sci..

[27] Visser S.N., Zablotsky B., Holbrook J.R., Danielson M.L., Bitsko R.H. (2015). Diagnostic Experiences of Children With Attention-Deficit/Hyperactivity Disorder. Natl. Health Stat. Rep..

[28] Barkley R.A. (2018). Attention-Deficit Hyperactivity Disorder: A Handbook for Diagnosis and Treatment.

[29] Richman J.E. (2016). The Developmental Eye Movement Test™ (DEM™).

[30] Martin N. (2017). Test of Visual Perceptual Skills.

[31] Beery K., Beery N. (2010). Test of Visual Motor Skills.

[32] Martin N.A., Brownell R. (2005). Test of Auditory Processing Skills–Third Edition (TAPS-3: SBE).

[33] Sonuga-Barke E.J.S., Becker S.P., Bölte S., Castellanos F.X., Franke B., Newcorn J.H., Nigg J.T., Rohde L.A., Simonoff E. (2023). Annual Research Review: Perspectives on progress in ADHD science–from characterization to cause. J. Child Psychol. Psychiatry.

[34] Cortese S., Kelly C., Chabernaud C., Proal E., Di Martino A., Milham M.P., Castellanos F.X. (2012). Toward systems neuroscience of ADHD: A meta-analysis of 55 fMRI studies. Am. J. Psychiatry.

[35] Barry R.J., Clarke A.R., McCarthy R., Selikowitz M., Rushby J.A., Ploskova E. (2004). EEG differences in children as a function of resting-state arousal level. Clin. Neurophysiol..

[36] Chen S., Sussman E.S. (2013). Context effects on auditory distraction. Biol. Psychol..

[37] Biederman J., Monuteaux M.C., Doyle A.E., Seidman L.J., Wilens T.E., Ferrero F., Morgan C.L., Faraone S.V. (2004). Impact of executive function deficits and attention-deficit/hyperactivity disorder (ADHD) on academic outcomes in children. J. Consult. Clin. Psychol..

[38] Martinussen R., Hayden J., Hogg-Johnson S., Tannock R. (2005). A meta-analysis of working memory impairments in children with attention-deficit/hyperactivity disorder. J. Am. Acad. Child Adolesc. Psychiatry.

[39] Kaufman A.S., Flanagan D.P., Alfonso V.C., Mascolo J.T. (2010). Essentials of Cross-Battery Assessment.

[40] Uddin L.Q., Kelly C., Biswal B., Xavier Castellanos F., Milham M.P. (2008). Functional connectivity in ADHD. Cogn. Brain Res..

[41] Zheng Y., Ye W., Korivi M., Liu Y., Hong F. (2022). Gender differences in fundamental motor skills proficiency in children aged 3–6 years: A systematic review and meta-analysis. Int. J. Environ. Res. Public Health.

[42] Barnett L.M., Lai S.K., Veldman S.L.C., Hardy L.L., Cliff D.P., Morgan P.J., Zask A., Lubans D.R., Shultz S.P., Ridgers N.D. (2016). Correlates of gross motor competence in children and adolescents: A systematic review and meta-analysis. Sport. Med..

[43] Gori S., Facoetti A. (2015). How the visual system develops and impacts learning: The role of visual-spatial attention and its influence on reading acquisition. Front. Psychol..

[44] Stein J. (2014). Dyslexia: The role of vision and visual attention. Curr. Dev. Disord. Rep..

[45] Birch E.E., Saunders R.A. (1992). Visual development and its clinical implications. Pediatr. Clin. N. Am..

